# A Rare Case of Pituitary Melanoma Metastasis: A Dramatic and Prolonged Response to Dabrafenib-Trametinib Therapy

**DOI:** 10.3389/fendo.2020.00471

**Published:** 2020-07-23

**Authors:** Marilda Mormando, Giulia Puliani, Agnese Barnabei, Rosa Lauretta, Marta Bianchini, Alfonsina Chiefari, Michelangelo Russillo, Francesco Cognetti, Luisa Romano, Marialuisa Appetecchia

**Affiliations:** ^1^Oncological Endocrinology Unit, IRCCS Regina Elena National Cancer Institute, Rome, Italy; ^2^Department of Experimental Medicine, Sapienza University of Rome, Rome, Italy; ^3^Division of Medical Oncology 1, IRCCS Regina Elena National Cancer Institute, Rome, Italy; ^4^Nuclear Medicine Unit, IRCCS Regina Elena National Cancer Institute, Rome, Italy

**Keywords:** melanoma, pituitary melanoma metastasis, pituitary, dabrafenib, trametinib, therapy

## Abstract

**Introduction:** Pituitary metastases (PM) are rare events and to date only very few cases of melanoma PM have been described in literature up to now.

**Case Presentation:** We describe the clinical history of a 33-year-old male patient who underwent surgical excision of an inter-scapular melanoma in 2008. The subsequent follow-up was negative for ~10 years. In September 2018, due to the onset of a severe headache, the patient underwent a brain magnetic resonance imaging, which showed an expansive mass in the saddle and suprasellar region with a maximum diameter of 17 mm. Pituitary function tests and visual field were normal. Worsening of the headache and the appearance of a left eye ptosis led the patient to surgical removal of the lesion in October 2018. The histological examination unexpectedly showed metastasis of the melanoma. Post-operative hormonal assessment showed secondary hypothyroidism and hypoadrenalism, which were both promptly treated, and a mild hypogonadism. Three months after surgery, a sellar MRI showed a persistent, increased pituitary mass (3 cm of diameter); fluorine-18-fluorodeoxyglucose positron emission tomography/computed tomography (^18^F-FDG PET/CT) detected an increased radiopharmaceutical uptake in the sellar region. Due to the persistence of the disease and the evidence of a BRAF V600E mutation, in February 2019, the patient underwent a combined treatment with dabrafenib (a BRAF inhibitor) and trametinib (mitogen-activated extracellular signal-regulate kinase inhibitor). Sellar MRI performed 6 months later showed no evidence of mass in the sellar region. The patient was in a good clinical condition and did not complain of headaches or other symptoms; there were no significant side-effects from the anticancer therapy. After 13 months of treatment, the patient showed no recurrence of the disease on morphological imaging. Anticancer therapy was confirmed, replacement therapies with hydrocortisone and levothyroxine continued and the pituitary-gonadal axis was restored.

**Conclusion:** This is a very interesting case, both for the rarity of the pituitary melanoma metastasis and for the singular therapeutic course carried out by the patient. This is the first case of a pituitary melanoma metastasis with BRAF mutation, successfully treated with the combination of dabrafenib and trametinib after incomplete surgical removal.

## Introduction

Pituitary metastases (PM) are a rare event; lung cancer is the most common cause among men (46%) while breast cancer accounts for half of the cases in women (1, 2), followed by renal, prostate and colon cancer (3–5% respectively), however, any type of tumor can metastasize in the pituitary region, including solid tumors and hematological malignancies (3). In the surgical series, PM represents <1% of patients undergoing transsphenoidal sellar mass surgery (1, 4). Melanoma metastases in the pituitary gland are extremely rare, with only a few reported cases. In 1857 L. Benjamin described, for the first time, a case of metastasis in the pituitary gland found during the autopsy of a patient with disseminated melanoma. Since then, only ten similar cases have been reported in the literature (5).

## Case Report

We report the case of a 33-year-old Caucasian man with a pituitary mass and a previous history of melanoma. In January 2008 the patient underwent surgical removal of a pigmented skin lesion of the interscapular region at our Institute. Histological examination showed a melanoma (IV Clark level, Breslow thickness 1.5 mm). In February 2008, the patient underwent an enlargement of the surgical wound and removal of bilateral sentinel lymph nodes, which were free from neoplastic infiltrations.

Thereafter the patient underwent clinical examination every 6 months for the first 5 years, and every 12 months after the first 5 years, without evidence of disease recurrence or new cutaneous lesions.

Ultrasound of the lymph node stations and of the peritumoral scar area were performed every 6 months and the abdominal ultrasound every 12 months in the following 10 years.

**Figure 1:**
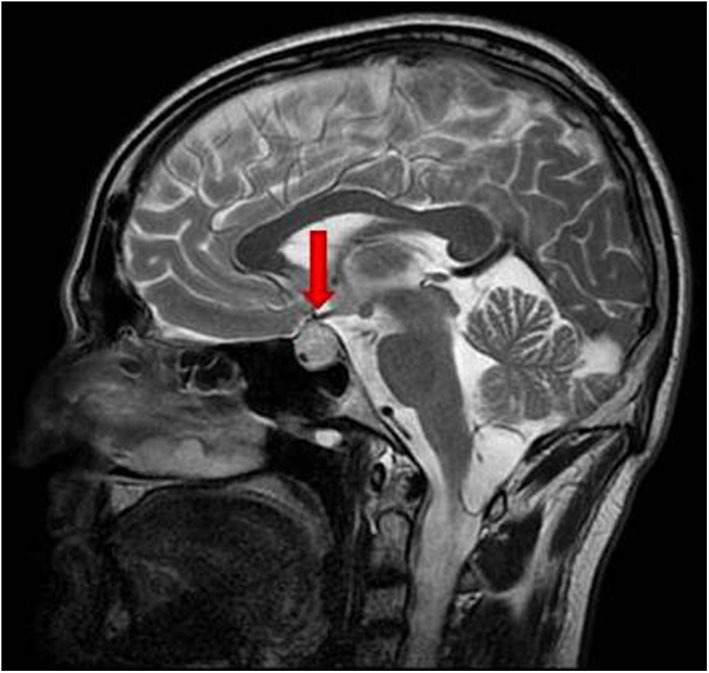
T2-weighted basal sagittal pituitary MRI performed at diagnosis. The red arrow indicates the sellar mass.

In August 2018, the patient presented with several severe headaches, not sensitive to common analgesic drugs. In September 2018 he performed a contrast-enhanced brain magnetic resonance imaging (MRI), which documented an expansive lesion in the left and suprasellar region, with a maximum diameter of 17 mm, hyperintense in T2-weighted sequences with an inhomogeneous contrast distribution due to the presence of internal colliquative areas (Figure 1). The left side of the mass was in contact with the optic chiasm. The initial radiological diagnosis was of pituitary macroadenoma. No visual field alterations were detected, and the pituitary hormone function tests were within the normal range. The patient was therefore candidate for elective surgery; however, after a few days he complained of a worsening of the headache and the appearance of a ptosis of the left eye. Brain computed tomography (CT) which was performed urgently 2 weeks after the MRI, documented two hyperdense areas, referred to as intralesional hemorrhages, in the pituitary mass.

In October 2018, the patient underwent surgical removal of the lesion using a trans-sphenoidal endoscopic approach. Post-operative hormonal tests performed few days later showed mild hypothyroidism and secondary hypoadrenalism for which hydrocortisone (20 mg daily) and levothyroxine (75 μg daily) oral therapy were promptly initiated, and a mild hypogonadism.

At histology, metastasis of the melanoma (maximum diameter 26 mm) was unexpectedly found. Neoplasm expressed vimentin, S100, MITF, HMB45, and melan-A; the specimen showed areas of necrosis, mitosis and focal brownish pigment.

**Figure 2:**
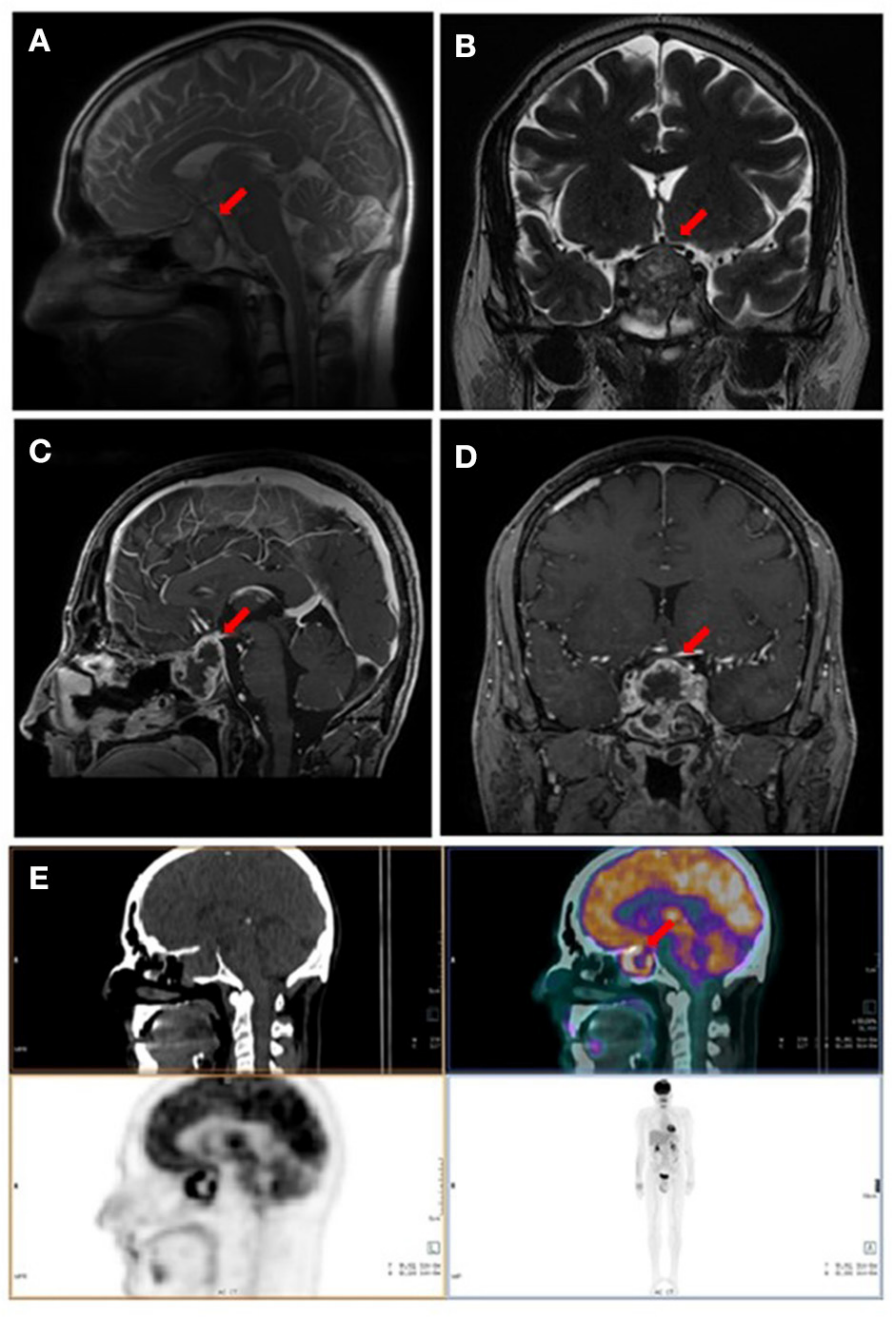
Imaging performed 3 months after surgery. **(A,B)** Pituitary MRI, T2-weighted basal sagittal and coronal sequences. **(C,D)** Pituitary MRI, T1- weighted post-gadolinium sagittal and coronal sequences. **(E)** Whole body fluorine-18-fluorodeoxyglucose positron emission tomography/computed tomography (^18^F-FDG PET/CT) showing pathological uptake in sellar region. Red arrows indicate the sellar lesion.

Due to a persistent headache, a sellar MRI was performed 3 months after surgery (January 2019), Imaging showed a persistent pituitary mass, characterized by increased size (maximum diameter of 3 cm), hypointense in T1-weighted and inhomogeneous in T2-weighted sequences, with a necrotic component (Figure 2).

In January 2019, a fluorine-18-fluorodeoxyglucose positron emission tomography/CT (^18^F-FDG PET/CT) was performed for restaging. An increased radiopharmaceutical uptake, associated with a necrotic area in the context, was documented in the sellar region. No other areas of increased uptake were identified (Figure 2).

Given the histological diagnosis and in order to decide the subsequent treatment, analysis of the BRAF gene mutation was carried out on the surgical specimen. After evidence of the BRAF V600E mutation and the persistence of disease, a schedule of a combined treatment was started in February 2019 with dabrafenib (a BRAF inhibitor, [BRAFi]) and trametinib (a mitogen-activated extracellular signal-regulated kinase, [MEK inhibitor]).

**Figure 3:**
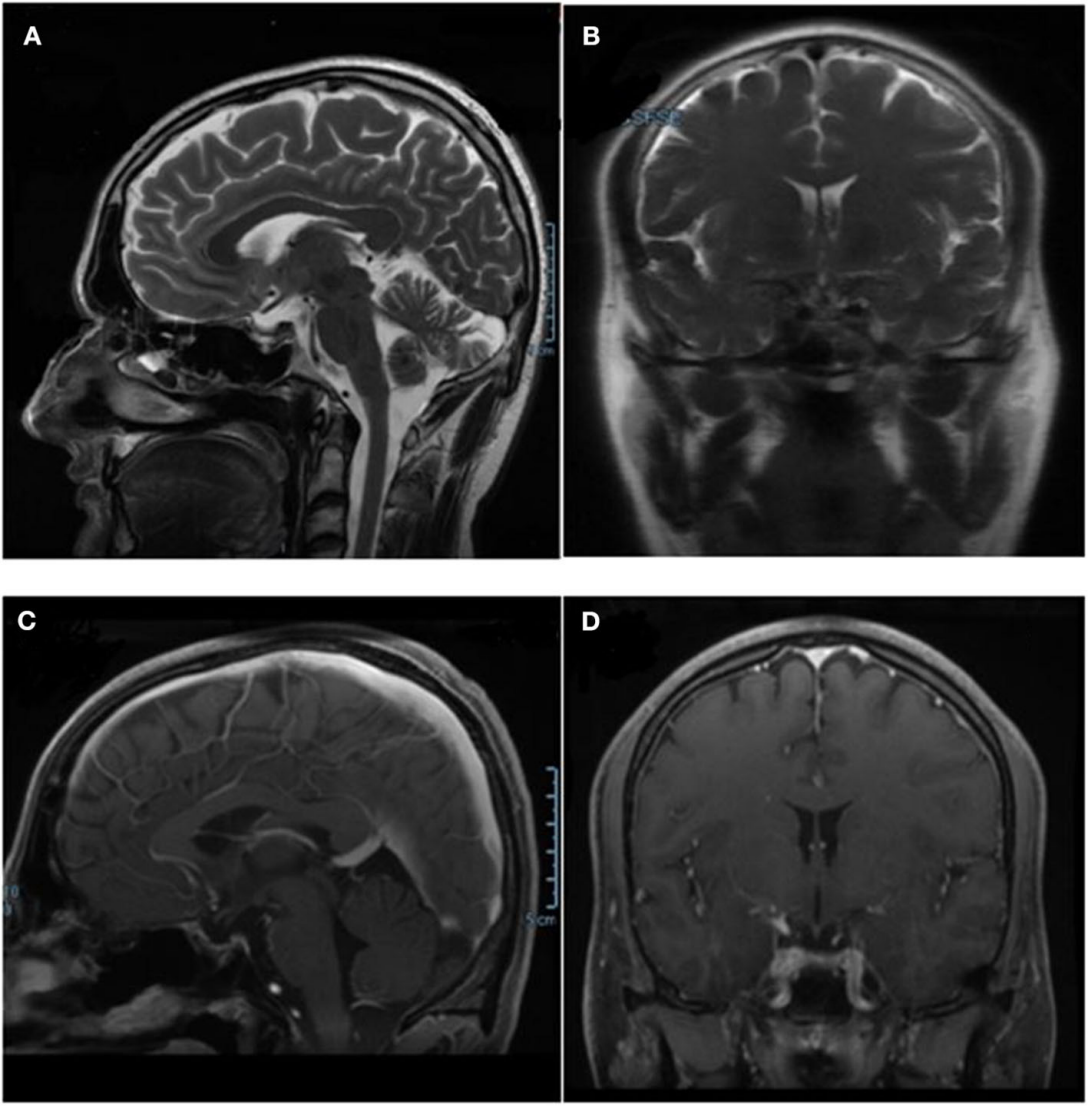
Pituitary MRI performed after combined therapy with dabrafenib and trametinib, showing the complete remission of the pituitary mass. **(A,B)** T2-weighted basal sagittal and coronal sequences. **(C,D)** T1- weighted post-gadolinium sagittal and coronal sequences MRI.

After 6 months of therapy, a pituitary MRI showed complete remission (CR) of the disease with no evidence of the mass in the sellar region (Figure 3). During therapy, the patient did not complain of headaches or other symptoms. He had no significant side-effects from the anti-cancer therapy. The pituitary-gonadal axis was restored while the recovery of pituitary-thyroid and pituitary-adrenal axes did not occur. FT4 levels were in normal range on levothyroxine therapy and morning ACTH and cortisol plasmatic levels were below the range after 24 h from last hydrocortisone administration. At the last follow-up visit, 13 months after the start of antineoplastic therapy, no evidence of persistence/recurrence of the disease was found at whole-body computerized axial tomography. Replacement therapy with hydrocortisone and levothyroxine was confirmed and antineoplastic therapy was continued.

## Discussion

Melanoma accounts for ~1% of all skin cancers; nevertheless, it is responsible for most skin cancer-related deaths. The worldwide incidence of melanoma has increased rapidly over the past 50 years (6). Its incidence is higher among fair-skinned populations and in low-latitude regions. Europe, North America, and Oceania are the most affected; about 85% of the world's annual cutaneous melanomas occur in populations from these regions. The incidence is higher among geriatric populations, reaching a peak at the seventh and eighth decades of life (7). This trend is found among most high-risk populations, including people in Australia, New Zealand and northern Europe (6). The incidence in the USA, however, peaks at the sixth decade of life. Although melanoma incidence is lower among people under the age of 40 years, melanoma is one of the most common cancer among teenagers and young adults. Overall, men are more susceptible to melanoma. This difference in incidence by sex is exemplified in the USA with an annual incidence of 29.2:100,000 cases in men compared with 17.3:100,000 cases in women (8, 9).

Although melanoma incidence has increased significantly over time, the 5-years survival rate has also increased. Survival rates largely depend on the stage of the disease at the time of diagnosis; the 5-year survival rate ranged from about 98% for localized disease to only about 23% for metastatic disease with previous traditional chemotherapy regimens (10). In early-stage cutaneous melanoma, although two-thirds of the relapses occurred within the first 2 years of diagnosis and nearly half of the first recurrences were locoregional metastasis, distant metastases can occur in the following 10 years from diagnosis. Furthermore, distant metastases alone and particularly brain metastases, although uncommon, are more frequent in men than women (11). Pituitary involvement is rare. To date, 10 cases of pituitary melanoma metastasis are described in literature, but only five of these are described in detail and have been confirmed by a histological examination (12–15). Table 1 summarizes the clinical features, onset symptoms, MRI, treatment, and outcomes of all patients with melanoma pituitary metastasis reported in literature to date. Cases of primary melanoma of the sellar region (16) and metastasis on preexisting pituitary mass (adenoma or oncocytoma) (17, 18) have been excluded from our analysis.

**Table 1:** Cases of melanoma metastases described in literature (12–15).

**References**	**Age**	**Sex**	**Onset symptoms**	**Pituitary function**	**Site of primary melanoma**	**Timing PM**	**MRI imaging at diagnosis**	**Other metastases**	**Treatment**	**Follow-up after surgery or biopsy**
Leung et al. (12)	46	M	DI	Not specified dysfunction	Neck	60 m	Size: NA MRI sequences: hyperintense T1, hypointense in T2 Cavernous sinuses involved: NA Chiasmal compression: NA	No	TS	FU: NA DFS: NA OS: NA
McCutcheon et al. (13) case 1	77	M	Diplopia	Hypogonadism	Chest wall	3 m	Size: >1 cm MRI sequences: NA Cavernous sinuses involved: yes Chiasmal compression: NA	No	TS, RT	FU: 6 m DFS: 6 m OS:UK
McCutcheon et al. (13) case 2	42	M	DI, temporal hemianopsia	Hypocortisolism Hypogonadism	Chest wall	72 m	Size: 25 mm MRI sequences: NA Cavernous sinuses involved: no Chiasmal compression: yes	Lung, retro-peritoneum	TS, RT, thalidomide, and temozolomide	FU: 17 m DFS: 0 m OS:6 m
Guzel et al. (15)	46	F	Headache	NA	Left shoulder	84 m	Size: 20 mm MRI sequences: isointense in T1 and T2 Cavernous sinuses involved: NA Chiasmal compression: NA	Cerebello pontine area	RT, temozolomide	FU: 17 m DFS: 0 OS:17
Masui et al. (14)	68	M	Anorexia, headache	Hypothyroidism	Stomach	PM was the first sign disease	Size: >1 cm MRI sequences: Hypointense in T2-weighted, inhomogeneous CE Cavernous sinuses involved: NA Chiasmal compression: yes	No	TS	FU: 0 DFS: NA OS:UK Patient refused treatment
Our case	33	M	Headache	Hypocortisolism Hypothyroidism Hypogonadism	Scapular region	127 m	Size: 17 mm MRI sequence: hyperintense in T2, inhomogeneous CE Cavernous sinuses involved: no Chiasmal compression: yes	No	TS BRAFi-MEKi	FU: 17 m DFS: 13 m OS:17 m

*M, male; MRI, magnetic resonance imaging; RT, radiotherapy; OS, overall survival; DI, diabetes insipidus; NA, not available; FU, follow-up; UK, unknown; m: months; TS, trans-sphenoidal surgery; DFS, disease free survival; CE, contrast enhancement*.

Three different metastatic modalities can be hypothesized for the metastatic spread of melanoma in the pituitary: (1) melanoma cells metastasize in the posterior pituitary through the inferior pituitary artery and therefore invade the anterior pituitary; (2) melanoma cells cross the blood-brain barrier of the adenohypophysis and invade the pituitary; and (3) melanoma cells spread through lymphatic microvessels and settle in the pituitary gland (14, 19).

Diabetes insipidus is present in about 50% of patients with PMs of all sites, in 25–45% of cases an anterior pituitary functional deficiency was reported, visual damage in 30%, ophthalmoplegia in 25%, and headache/retroorbital pain in 20% of cases was reported (3, 20, 21). In pituitary melanoma metastases, the clinical presentation is the same as that observed in the case of PMs due to other neoplasms: the onset symptoms are diabetes insipidus, headache, visual problems while pituitary dysfunctions include hypothyroidism, hypocortisolism, and hypogonadism (Table 1).

Pituitary MRI in all PMs usually reveals a non-homogeneous invasive sellar mass and loss of posterior bright spot. Sometimes it is difficult to differentiate PMs radiologically from adenomas or other sellar lesions; the presence of sellar bone erosion without sellar enlargement may suggest diagnosis of PM (3). At MRI, melanoma metastasis, due to melanin storage, appears with a typical hyperintensity on basal T1-weighted sequences and hypointensity on basal T2-weightedsequences; in our case pituitary imaging was in conflict: the mass was hyperintense in T2 sequences, suggesting pituitary adenoma, but the heterogeneity due to the intralesional bleeding suggested a PM.

All patients with PMs at all sites have an unfavorable prognosis, and usually most of them die within 12 months after diagnosis, more often owing to the progression of the primary malignancy (20). In recent years, a series of patients with PM showed an improved prognosis with a an extended median survival, probably due to the use of a multimodal treatment approach, including pituitary surgery, sellar radiotherapy, hormonal replacement and chemotherapy (22). The few cases, in literature, describing melanoma PM had an unsatisfactory prognosis or short disease-free survival (12–14). In our case, however, we observed a disease-free survival of 13 months thanks to the combined treatment with dabrafenib-trametinib and an overall survival (OS) of 17 months.

Monoclonal antibodies that block programmed cell death receptor 1 (PD-1) as pembrolizumab or nivolumab, are the first therapeutic option in advanced/metastatic melanoma (23, 24). These drugs could cause swelling and inflammatory reaction in the tumor area potentially leading to compressive symptoms and pituitary deficiencies. Furthermore, the PD-1 inhibitors could cause hypophysitis (much more rarely than monoclonal antibody against the cytotoxic T lymphocyte antigen-4 [CTLA-4] as ipilimumab) with a maximum incidence of 1.2% for pembrolizumab and 0.9% for nivolumab (25, 26). The compressive effect due to the mass swelling and the possible pituitary deficiencies could be managed, respectively, by high dose corticosteroids and hormonal replacement therapies.

Considering that 50% of patients with metastatic melanoma have mutations in BRAF, located in BRAF exon 15 at V600 in 95% of cases (27), an additional therapeutic option is BRAFi. The approval of BRAFi vemurafenib in 2011 and dabrafenib in 2012 by the US Food and Drug Administration (FDA) led to substantial improvements in progression-free survival (PFS) and OS compared with therapies available at that time (28, 29). BRAF is a member of the RAF kinase family, with a role in the ERK/mitogen-activated protein kinase (MAPK) pathway, a signaling cascade that regulates cell proliferation, differentiation and survival. The clinical response observed with BRAFi is, however, limited by acquired resistance to these agents through the reactivation of the MAPK pathway via transactivation of RAF and subsequent MEK/ERK phosphorylation in cells with wild-type BRAF (30).

The addition of a MEKi, such as trametinib or cobimetinib, to BRAFi mitigates a pathway of resistance, increasing response rates with an improved OS, without significant cumulative toxicity. Dual inhibition of the MAPK pathway with the addition of MEKi therapy to BRAFi therapy has been demonstrated in subsequent clinical studies aimed at further improving efficacy results and reducing the toxicities associated with reactivation of the MAPK pathway, including the incidence of secondary malignancies (31). A scheduled treatment with both BRAFi and MEKi showed a better efficacy overall compared to BRAFi monotherapy (32). Three BRAFi- MEKi combination treatments (dabrafenib-trametinib, vemurafenib-cobimetinib, and encorafenib-binimetinib) are currently considered the standard treatments for patients with advanced BRAF-mutant melanoma (33–35).

In a recent landmark analysis, the 5-year OS in patients with BRAF V600 mutant metastatic melanoma treated with dabrafenib-trametinib was 28% with a 76% response rate and CR observed in 17% of patients. In patients with CR, the 3-year PFS rate was 67, 40% at 5 years and the median PFS was 39.6 months (36). Furthermore, a case of long-term complete response 18 months after treatment discontinuation, has been reported (37).

We report the first case, in literature, of a histologically proven melanoma PM successfully treated with the combination of dabrafenib-trametinib. Considering the uncommon site of melanoma metastasis and the long timeframe between the primary disease and the recurrence, a multidisciplinary team reassessed the case. The histological evaluation revealed focal brownish pigments in the removed sellar mass suggestive of melanoma metastasis. A careful clinical and dermatological examination was conducted to rule out the presence of new cutaneous lesions suspected for melanoma. No further immunohistochemical analysis were available to ensure the relationship between primary and metastatic tumor. At the time of disease recurrence after surgical intervention, the presence of the BRAF mutation in our patients has allowed the use of dabrafenib-trametinib, an effective anticancer therapy not burdened by side-effects on the pituitary gland, already compromised by the disease.

It would be very interesting to understand the mechanisms behind the rapid and complete response to the combination therapy with dabrafenib-trametinib. Some studies reported that, in metastatic melanomas, the mechanisms involved in the response to dabrafenib therapy may involve the pituitary tumor-transforming gene-1 (PTTG1), an oncogene overexpressed in a variety of cancer cell lines as well as in a wide range of primary and metastatic tumors. PTTG1 is also overexpressed in a variety of endocrine-related tumors, in particular, pituitary, thyroid, breast, ovary, and uterus tumors (38). In a recent study, the authors demonstrated that in dabrafenib-sensitive cells, inhibition of cell growth and extra cellular matrix invasion, occur, at least in part, through the down regulation of PTTG1 expression (39). In our case, we could assume a downregulation of the PTTG1 oncogene, mediated by the BRAFi, which could be strongly expressed by the tumor; it has also been observed in animal models of pituitary tumors (40).

## Conclusions

PMs often represent the manifestation of systemic malignancies and had an unfavorable prognosis. Literature confirmed that pituitary melanoma metastases represent a very rare event burdened by therapeutic failure. Our case report suggests that in metastatic melanomas, the new antineoplastic drugs acting on the BRAF system could represent an effective therapy and a real chance of cure with prolonged disease-free survival, without affecting quality of life, an important issue especially in young patients.

## Data Availability Statement

The original contributions presented in the study are included in the article/supplementary material, further inquiries can be directed to the corresponding author/s.

## Ethics Statement

A written informed consent was obtained from the patient for the publication of any potentially identifiable images or data included in this article.

## Author Contributions

MM wrote the manuscript. GP contributed to the manuscript preparation. RL, MB, and AC contributed to the concept and design for the study. MR and FC conducted the oncological management and follow up. LR contributed to the performing and interpretation of nuclear imaging. AB and MA supervised this work. All authors reviewed and approved the final version of the manuscript.

## Conflict of Interest

The authors declare that the research was conducted in the absence of any commercial or financial relationships that could be construed as a potential conflict of interest.
